# Understanding Players’ Sportspersonship Attitude, Expectancy-Related Beliefs, and Subjective Task Values in Field Hockey: An Integrated Approach

**DOI:** 10.3390/ijerph19084819

**Published:** 2022-04-15

**Authors:** Kanagarajah Rarujanai, Eng Wah Teo, Chin Ngien Siong, Arthur Ling, Garry Kuan

**Affiliations:** 1 Centre for Sport and Exercise Sciences, University of Malaya, Kuala Lumpur 50603, Malaysia; kanagarajah@ipgmktar.edu.my (K.R.); ling_wei87@hotmail.com (A.L.); 2 Institute of Teacher Education, Batu Lintang Campus, Kuching 93200, Malaysia; ngiensiong@gmail.com; 3 Exercise and Sports Science Programme, School of Health Sciences, Universiti Sains Malaysia, Kubang Kerian 16150, Malaysia

**Keywords:** sportspersonship attitude, expectancy beliefs, subjective task values, field hockey

## Abstract

(1) Background: The purpose of this study was to examine the relationship between expectancy-value components and attitudes toward sportspersonship among Malaysian adolescent field hockey players. This study also examined the effect of expectancy beliefs, task values, and sportspersonship attitude on the motivation of adolescent field hockey players by gender and age group. (2) Methods: The Malay versioned Expectancy Value Model Questionnaire and the Malay versioned Multidimensional Sportspersonship Orientations Scale were administered on 730 respondents (µ = 15.46 ± 1.83 years). (3) Results: The expectancy values and attainment value (r = 0.894), utility value and attainment value (r = 0.833) were highly correlated. There was no significant gender difference in expectancy, task values, and sportspersonship attitude dimensions. The main effect of age group was significant on task values: *F* (2724) = 4.19; *p* = 0.01. The difference was indicated between age groups of 15–16 years and 12–14 years (*p* = 0.02, *d* = 0.014) under task values variable. (4) Conclusions: There is no significant relationships between sportspersonship attitude (MSOS-M) and of expectancy beliefs and task values (EVMQ-M). To conclude, female and younger players demonstrate lower expectancy beliefs, task values, and sportspersonship attitudes than male and older field hockey players.

## 1. Introduction

In general, the act or attitude of sportspersonship (sportsmanship) provides an ex-tensive insight into sports participation involving interpersonal aspects and against win-at-all-costs approach [1]. From a psychological point of view, the concept of sportspersonship is strongly associated with practice and development of good moral acts [2]. According to Karatas and Savas [3], this character developing element in sport has developed as an integral part of sporting culture, reflecting the moral life of an individual.

The moral reasoning and motivational intention (extrinsic motivation) involving sportspersonship attitudes differ according to the individuals and circumstances. Vallerand et al. [3] proposed five multidimensional constructs of sportspersonship: total commitment to sport participation, adherence to social conventions, respect and concern for rules and officials, respect and concern for opponents, and a negative attitude toward sportspersonship. The definition of sportspersonship varies according to sport and circumstance [3].

As a means to study individuals’ behavioral motivations, the expectancy value theory has been commonly used by researchers [4,5]. Expectancy beliefs and task values can have a significant impact on the players’ decision making in achievement-related situations [6]. Expectancy beliefs encompass both success expectations and belief in one’s ability. The expectation of success is defined as an individual’s belief in their ability to engage in and complete a given task, while an ability-related belief is defined as individuals’ self-evaluation towards their ability and competency in completing given tasks [7]. Likewise, task values are interchangeably an important variable under the expectancy value model related to affective memories [8]. There are mainly four components under task values: attainment, intrinsic, utility, and cost.

The attainment value, known as importance, refers to the core personal value or self-image one associates regarding expected performance on a task or activity, while intrinsic motivation deals with the pleasure and [9] enjoyment experienced by an individual by performing a given task or activity [10]. Utility value or usefulness explains how an individual view his or her current task or activity in relation to his or her current or future goals [10], whereas cost value refers to the negative values students assign to a particular task, such as time and energy spent on other options or choices [7]. Numerous researchers who used an expectancy value model in their study did not place a high premium on cost value. This could be due to the negative significances it can bring to both task values and expectancy beliefs overall [7,9]. Taking that into consideration, this study will also omit this component based on similar empirical reasons.

**Figure 1:**
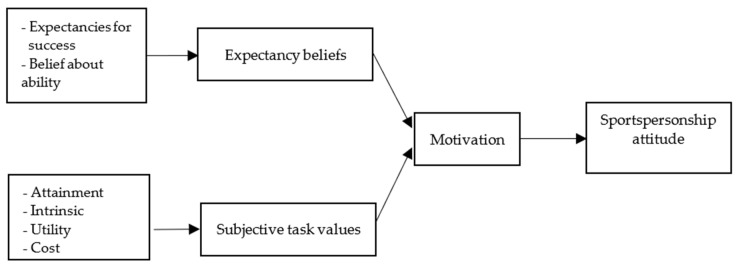
Conceptual framework of the expectancy beliefs and subjective task values towards sportspersonship attitudes.

Generally, the expectancy value theory reflects one’s behavior, which is associated with the choice of achievement and performance of the task. The theory posits the analyzing of motivational intention to predict the engagement of one’s behavior, which is the engagement of unsportsmanlike behavior in their pursuit of triumph and excellence in performance [10]. The multidimensions of sportspersonship also pave a path in which sportspersonship can interact with achievement motivation in terms of their expectancy beliefs and subjective task values, which can influence behavioral outcomes [6] (Figure 1). 

Chan et al. [11] in their study incorporated expectancy belief values in the context of social-cognitive components of doping avoidance. Adell et al. [12] conducted a similar study incorporating beliefs, values, and attitudes among basketball players. The study revealed that personal beliefs are transferred to values in sport and, in turn, are related to attitudes as the game progresses. Task-oriented players tend to demonstrate more prosocial attitudes in sport, while ego-oriented players exhibited greater antisocial attitudes in sport [12]. However, based on a thorough review of the literature, little is known about the relationship between expectancy-value components and sportspersonship attitudes in field hockey. This relationship has never been investigated in Southeast Asia and particularly in Malaysia in either sporting or non-sporting contexts.

This study will be beneficial in the development of more effective and practical guidelines for motivating and maintaining adolescent players’ positive beliefs and values in field hockey participation. This will indirectly assist the players in dealing with their evaluation of self-ability and competence in the sport. On the other hand, this study will assist the coaches in determining their players’ values towards the sport, especially in Malaysian settings.

In this regard, we aim to explore the relationship between expectancy-value components and sportspersonship attitude among Malaysian adolescent field hockey players. Secondly, we also aim to examine the effect of expectancy beliefs, task values, and sportspersonship attitude on adolescent field hockey player’s motivation based on gender and age groups differences.

## 2. Materials and Methods

### 2.1. Participants

Table 1 showed the demographic characteristics of the participants. A total of 730 adolescent field hockey players (384 males, 346 females) aged between 12 and 19 years (M = 15.46 ± 1.83 years) from Malaysia participated in this study. The participants were adolescent players who participated in Malaysian Junior Hockey League organized by the Malaysian Hockey Confederation (MHC), MSSM field hockey competitions at state and national levels, field hockey development programs by state sports councils, and 1 Mas Program under MHC. Participants were divided into three different age groups; 229 participants were from Under-19 (17–19 years old), 264 participants were from Under-16 (15–16 years old), and 237 were participants from Under-14 (12–14 years old). The Krejcie and Morgan [13] sample size determination table was used to calculate the sample size.

### 2.2. Measures

#### 2.2.1. Malay Versioned Expectancy Value Model Questionnaire (EVMQ-M)

Eccles et al. [6] developed the original English version of the EVMQ, which consisted of 11 items for assessing players’ or sport users’ expectancy-related beliefs (5 items), attainment value (2 items), intrinsic value (2 items), and utility value (2 items). The numbers of items were maintained in the Malay version (EVMQ-M). Meanwhile, construct validity based on model fit indices for both the questionnaires revealed good data fit. EVMQ-M revealed a data fit of χ^2^ (*df* = 39) = 84.07, CFI = 0.97, GFI = 0.94, TLI = 0.95, and RMSEA = 0.07, ChiSq/*df* = 2.16, whereas the overall Cronbach’s alpha coefficient of EVMQ-Malay is 0.89, while the alpha coefficients for four subscales (expectancy belief, attainment, intrinsic, and utility) ranges from 0.70 to 0.87.

#### 2.2.2. Malay Versioned Multidimensional Sportspersonship Orientations Scale (MSOS-M)

The number of items of the original English version MSOS (25 items) by Vallerand et al. [7] were reduced to 19 items in the Malay version (MSOS-M) [14] due to factor loading considerations of below 0.60. MSOS-M is composed of five distinct sportspersonship orientations: respect for social conventions (4 items), respect for rules and officials (3 items), respect for one’s full commitment to sport participation (4 items), respect and concern for the opponent (4 items), and a negative attitude toward the practice of sport (4 items) (4 items). MSOS is based on a 5-point Likert scale from ‘Doesn’t correspond to me at all (1)’ to ‘Corresponds to me exactly (5)’. MSOS-M revealed a data fit of χ^2^ (*df* = 146) = 321.76, CFI = 0.92, GFI = 0.87, TLI = 0.90, RMSEA = 0.07, and ChiSq/*df* = 2.20. In addition, the overall Cronbach’s alpha value for all five subscales in MSOS-M [14] is 0.84, while individual subscale alpha ranges from 0.71 to 0.82. A separate set of demographic questions: gender, age, date of birth, race, school/institution address, and level of sport participation was included to obtain the information about participants.

### 2.3. Procedures

The participants in this study were chosen through a random cluster sampling technique from the Malaysian Hockey Confederation (MHC)’s national junior hockey league. This study obtained approval from the University Malaya Research Ethics Committee (UMREC) prior to data collection (Reference Number: UM.TNC2/UMREC-212). Participants under the age of 18 provided written parental consent, while those over the age of 18 signed the consent form. Participants were informed about the study’s objectives and their rights as a participant. They were reminded that they should respond to questions only if they felt comfortable doing so. Participants were free to withdraw at any time and were told that their withdrawal would not result in any penalty.

### 2.4. Statistical Analysis

SPSS versioned 27.0 (SPSS Inc., Chicago, IL, USA) and AMOS versioned 23.0 (Amos Development Corporation, Meadville, PA, USA), were used for data analysis in computing the descriptive analysis, correlation analysis, factorial analysis, reliability analysis, multiple comparisons (Bonferroni’s test), and regression analysis. Gender differences were compared separately using independent *t*-test. Two-way ANOVA was used to examine the age groups differences. The level of significance was set at alpha *p* < 0.05. The effect size (ES) was later computed and categorized based on Cohen’s (1988) recommendations.

## 3. Results

The descriptive statistics for expectancy beliefs, task values, and sportspersonship attitude were presented. The reliability analysis of expectancy-related beliefs and subjective values using EVMQ-M was presented first, followed by the differences in sportspersonship attitude variables using MSOS-M. Gender, age group, and locality differences were presented accordingly. Regression analysis examining the relationships among variables between the expectancy-value model and achievement goal theory was conducted at the end.

### 3.1. Descriptive Statistics for Expectancy Beliefs, Task Values & Sportspersonship Attitude Factors

Table 2 shows the descriptive statistics of each factor included in EVMQ-M and MSOS-M. Firstly, for reliability values (Cronbach’s alpha coefficient), all factors were over 0.70, with the exception of intrinsic and utility values, which had slightly lower values (0.68 and 0.63). However, according to Hair et al. [15] and Chai et al. [16], this internal validity could be acknowledged due to the factors that consist of smaller number of items as well as the different attributes of the samples. Next, means and standard deviations of each factor were calculated. Regarding the means, higher values were obtained for attainment value (M = 5.82 ± 1.20) under EVMQ-M and respect for social conventions factors (M = 4.14 ± 0.62) under MSOS-M, whereas the utility value had a low mean (M = 5.55 ± 1.10) for EVMQ-M and negative approach toward the practice of sport factor had a low mean (M = 2.31 ± 0.90) for MSOS-M.

### 3.2. Correlation between EVMQ-M and MSOS-M

Table 3 shows the relationship between expectancy-beliefs, task values components (utility, attainment, and intrinsic), and sportspersonship attitude components (respect for social conventions, respect for the rules and the officials, respect for one’s full commitment, and respect and concern for the opponent and negative approach toward the practice of sport). The scores of expectancy values and attainment value (r = 0.894) and utility value and attainment value (r = 0.833) were highly correlated. This was followed by the score of expectancy values and intrinsic value (r = 0.795), expectancy values and utility value (r = 0.771), intrinsic value and attainment value (r = 0.747), and finally interactions between intrinsic value and utility value (r = 0.715). A few moderate correlation values had been recorded among the variables studied: respect and concern for the opponent vs. respect for social conventions (r = 0.668); respect and concern for the opponent vs. respect for the rules and the officials (r = 0.659); respect for the rules and the officials vs. respect for social conventions (r = 0.621); respect and concern for the opponent vs. respect for one’s full commitment (r = 0.608); negative approach toward the practice of sport vs. respect and concern for the opponent (r = −0.528); respect for one’s full commitment vs. respect for the rules and the officials (r = 0.480); and respect for one’s full commitment vs. respect for social conventions (r = 0.461). Three other relationships between variables studied were recorded as poorly correlated: negative approach toward the practice of sport vs. respect for the rules and the officials (r = −0.381); negative approach toward the practice of sport vs. respect for one’s full commitment (r = −0.369); and negative approach toward the practice of sport vs. respect for social conventions (r = −0.333). Other studied variables under EVMQ-M and MSOS-M showed no significant correlation between each other.

### 3.3. Gender Differences

Table 4 showed that there was no significant gender difference in the expectancy beliefs (M = 4.94 vs. 4.87; *p* = 0.46), task values (M = 5.10 vs. 4.94; *p* = 0.12), and sportspersonship attitude (M = 3.71 vs. 3.68; *p* = 0.39) dimensions. The *p*-value for all three factors recorded more than 0.05, indicating that there was no significant gender differences among the participants. Overall, male players showed higher total scores for all three factors compared to female players.

A one-way analysis of variance (ANOVA) was computed for participants’ age group of expectancy beliefs, task values, and sportspersonship attitude in field hockey participation (Table 5). The main effect of field hockey participation on expectancy values was not significant: *F* (2727) = 0.63; *p* = 0.53. Participants aged 17–19 years old were more determined towards the expectancy beliefs dimension (M = 4.97, SD = 1.31) compared to 15–16 years old participants (M = 4.91, SD = 1.31) and 12–14 years old participants (M = 4.83, SD = 1.42). The main effect of field hockey participation on task values was significant: *F* (2727) = 4.19; *p* = 0.01. Participants aged 15–16 years old attributed the task values dimension as more valuable (M = 5.15, SD = 1.36) than 17–19 years old participants (M = 5.07, SD = 1.33) and 12–14 years old participants (M = 4.82, SD = 1.33). Meanwhile, the main effect of field hockey participation on sportspersonship attitude was not significant, *F* (2727) = 0.94, *p* = 0.94. Participants aged 12–14 years old showed the least purpose in practicing sportspersonship attitude (M = 3.67, SD = 0.43) compared to participants aged 17–19 years old (M = 3.71, SD = 0.40) and 15–16 years old (M = 3.71, SD = 0.41).

### 3.4. Age Group Differences

A post hoc test for task values variables shown in Table 6 revealed that there was a significant difference between age groups 15–16 years and 12–14 years (*p* = 0.02, *d* = 0.014, small effect size) under task values variable.

Table 7 revealed Bonferroni-adjusted comparisons and indicated that there were only two statistically significant differences recorded in term of pairwise comparison of gender and the age groups. The age group of 12–14 years rated male athletes 0.34 points higher in expectancy beliefs than the female athletes (*p* = 0.05, 95% CI of the difference = −0.01 to 0.69), while the age group of 12–14 years also rated the male athletes 0.42 points higher in task values than the female athletes (*p* = 0.02, 95% CI of the difference = 0.07 to 0.77). Sportspersonship attitude revealed no statistical differences between the pair.

## 4. Discussion

The purpose of this study was to gain a better understanding of the relationship between sportspersonship and expectancy-value components in predicting achievement-related outcomes. In order to provide a better understanding of the relationship between sportspersonship and expectancy-value components in predicting achievement-related outcomes, we examined the relationship between expectancy-value components and sportspersonship orientation in a sample of Malaysian adolescent field hockey players. Pearson’s correlation coefficient data analyses revealed that almost all the variables of sportspersonship orientations and expectancy-value components are positively related to each other (Table 3), except for respect for social convention vs. expectancy beliefs (r = −0.002); respect for one’s full commitment vs. expectancy beliefs (r = −0.014); respect for one’s full commitment vs. utility (r = −0.023); respect for social conventions vs. utility (r = −0.012); negative approach toward the practice of sport vs. respect for the rules and the officials (r = −0.381); negative approach toward the practice of sport vs. respect for one’s full commitment (r = −0.369); and negative approach toward the practice of sport vs. respect for social conventions (r = −0.333).

This study revealed that expectancy values and attainment value (r = 0.894) and utility value and attainment value (r = 0.833) were highly correlated. Attainment and utility values as major contributors under task value dimension are observed as greatly influencing expectancy beliefs among the players. This result was justified by a study conducted by Chin, Teo, Kuan, and Yi [17], which found positive and good relationships between expectancy beliefs and task values (r = 0.78). In another similar study, Xiang, McBride and Guan [18] also had shown evidence of good correlations between both variables and justified the greater influence both variables have on each other. This implies that athletes who have higher beliefs in sports would attribute the sport for greater values.

Meanwhile, it was found that the utility value and attainment value showed greater correlation too as utility and attainment values often do not factor as separate scales. Conley [19] discovered that participants’ beliefs about the utility and attainment values of the subject were the same in her study involving 1870 math classroom students. Previously, utility and attainment value items have been merged under one subscale, and studies have reported separate interest values and combined utility/attainment values [20]. This indicates that both utility and attainment values reflect how extrinsically an athlete can observe the existence of sports in their daily life as a whole.

This study also examined the effect of expectancy beliefs, task values, and sportspersonship attitude on adolescent field hockey player’s motivation and the relationship between gender and different age groups. Our results revealed that male athletes demonstrated higher expectancy beliefs, task values, and sportspersonship attitudes than female athletes in field hockey (Table 4). These results strongly support previous studies [5,8] that reported boys demonstrate higher expectancy-related beliefs than girls in their sports performance. Additionally, Jacobs et al. [13] discovered that boys have stronger competence beliefs and task values in sports from elementary school to high school. Numerous researchers [5,13] hypothesized that these gender differences could be explained by participation in gender-appropriate activities, which increase expectancy beliefs and task values. For instance, gender differences have been observed more frequently in gender preference sports [14], such as soccer or volleyball, because girls and boys frequently value activities perceived to be gender appropriate. From this vantage point, the distinction between boys and girls is unsurprising.

Following this, we discovered no significant difference in attitudes toward sportspersonship between genders (Table 4). Our finding is consistent with Rahimizadeh et al.’s [21] investigation of the relationship between performance and aggression attitude. They discovered no significant difference between males and females in any aspect of sportspersonship, including invasion, violence, and stubbornness. Generally, male athletes practice lower sportspersonship attitude in sport, exhibiting their dominance of sport [8]. However, female athletes are no exception in sports too [22]. This is especially true for higher level competition or elite sports in terms of winning at all cost situations. In another related study, Lidor and Ziv [23] found that there was not much difference from the way women field hockey players were familiarized with the sport, learning the norms and loopholes. Female athletes display eagerness and become more competitive and professional, which is equal to male athletes, by demonstrating recognized male attitudes in field hockey including unsportspersonship attitude. This could sum up the reason for no significance differences found between genders in term of sportspersonship attitude.

From the perceptive of age groups, players aged 17–19 years old demonstrated higher mean values (4.97 ± 1.314) in expectancy beliefs (Table 5). Our result is consistent with Gurpinar and Kursun [24] who reported that older athletes experience greater expectancy beliefs than younger athletes. This could be because players in this age-group could have probably played field hockey for at least more than 4 years and are aware of the competency levels that the sport requires at higher stages of competition. They are at the last stage of junior level hockey before venturing into senior or elite level hockey soon, and they have their confidence level at topmost level compared to the other younger age groups [25].

On the other hand, the players aged 15–16 years old showed greater task values in comparison to the other age groups. In a previous study by Studer and Knecht [26], they found that middle-aged athlete’s perceptions of subjective task values were more positive than the beliefs of other age-group athletes. In Malaysia, active sport participation is regarded as an additional requirement to pursue intended tertiary level education. Being active in sport at the age of 15–16 years is believed to be important to maintain or progress to greater level of participation in order to ensure a guaranteed extracurricular accomplishment upon graduating from secondary schools.

In this study, no statistical difference was found in the sportspersonship attitude of the adolescent field hockey players in relation to their age groups (Table 5). Our findings are in line with those other similar studies [27,28]. Secondary school students who participate in sports demonstrated higher values and sportspersonship [28] than elite players. This could be possibly due to sociocultural settings, sample population, motivational climate, and advancement in technology [27]. This is due to the circumstances whereby elite players play at elite tournaments, i.e., Olympics or World Cup, with higher prestige and circumstances that are more at stake compared to secondary school students playing at school or state-level tournaments [20,29].

Our results also showed significant differences between age groups of 15–16 years and 12–14 years groups under task-value variables (Table 6). In Malaysia, most of the under 14-year-old players are not required to sit for any national public examination, with no special requirements needed for them to progress to the next levels of education. Indirectly, this could result them to regard the sport more seriously and instill more importance in the sport, in addition to spending more time engaging with the sport. However, in a separate study by Gurpinar and Kursun [24], they observed significant differences between age groups (team sports) in the component of ‘respect for one’s full commitment toward sport’ and ‘respect and concern for the opponent’. The different results could be due to different sport environment, different type of sports, different rules, field settings, and target set for winning, even though both involved team sports [24,29].

The findings of this study examined adolescent hockey players’ beliefs, values, and sportspersonship attitude toward sports by age groups and gender, as well as create enthusiastic youths at national and international level. This study will be beneficial to formulate more effective and practical guidelines in motivating and maintaining adolescents’ positive beliefs in field hockey participation.

## 5. Conclusions

It was found that there were no significant relationships between EVMQ-M and MSOS-M, which revealed that hockey players’ perceptions may not completely reflect their understanding of sportspersonship with expectancy beliefs and task values. Therefore, assessing sportspersonship variables on expectancy beliefs and values is deemed crucial for better performance, which could be further examined. Based on the outcome of this study, it is advisable that future research studies utilise both instruments separately for its optimum benefit.

In comparison to their older counterparts, younger Malaysian field hockey players demonstrated a weaker sense of sportspersonship integrated with their beliefs and values. Additionally, male and female athletes exhibit no differences in their approach to practice sportspersonship in field hockey. As a result, we proposed that sportspersonship-focused programs be implemented at an early age, beginning with all local junior sports programs and even during physical education classes, in order to foster positive attitudes and sportspersonship attitudes among athletes.

## Figures and Tables

**Table 1:** Demographic characteristics of the participants.

Variables	*N* (%)
Gender	
Male	384 (52.6)
Female	346 (47.4)
Location (Zones)	
Northern	147 (20.1)
Central	157 (21.5)
Southern	156 (21.4)
Eastern	151 (20.7)
Age Group	* 15.46 ± 1.83
17–19 years old	229 (31.4)
15–16 years old	264 (36.2)
12–14 years old	237 (32.5)

* Data presented as mean ± standard deviation.

**Table 2:** Descriptive statistic results of expectancy beliefs, task values, and sportspersonship attitude factors (*n* = 730).

	Mean (M)	SD	α
EVMQ-M			
Overall	5.64	1.02	0.8
Expectancy belief	5.6	0.8	0.77
Task values	5.65	0.89	0.8
Attainment	5.82	1.2	0.75
Intrinsic	5.59	0.98	0.68
Utility	5.55	1.1	0.63
MSOS-M			
Overall	5.64	0.71	0.72
Respect for social conventions	4.14	0.62	0.79
Respect for the rules and the officials	4.07	0.65	0.8
Respect for one’s full commitment	4.12	0.68	0.79
Respect and concern for the opponent	4.01	0.72	0.83
Negative approach toward the practice of sport	2.31	0.9	0.87

Note. SD = Standard Deviation; α = Cronbach’s alpha.

**Table 3:** Correlation between Expectancy Value Model Questionnaire Malay (EVMQ-M) and Multidimensional Sportspersonship Orientations Scale-Malay (MSOS-M).

Variables	1	2	3	4	5	6	7	8	9	M	SD
1. Expectancy belief										4.90	1.34
2. Utility	0.771 **									5.04	1.31
3. Attainment	0.894 **	0.833 **								4.96	1.43
4. Intrinsic	0.795 **	0.715 **	0.747 **							5.06	1.64
5. Respect for social conventions	−0.002	−0.012	0.003	0.008						4.18	0.69
6. Respect for the rules and the officials	0.029	0.014	0.034	0.050	0.621 **					3.96	0.77
7. Respect for one’s full commitment	−0.014	−0.023	0.005	0.002	0.461 **	0.480 **				4.22	0.67
8. Respect and concern for the opponent	0.073 *	0.015	0.061	0.067	0.668 **	0.659 **	0.608 **			4.03	0.79
9. Negative approach toward the practice of sport	0.005	0.042	0.008	0.035	−0.333 **	−0.381 **	−0.369 **	−0.528 **		2.15	0.98

* *p* < 0.05; ** *p* < 0.01.

**Table 4:** Expectancy beliefs, task values, and sportspersonship attitude according to gender.

Subscales		Mean	SD	*t*-Test	*p*-Value	Effect Size *(d)*
Expectancy beliefs	Male	4.94	1.34	0.738	0.46	0.001
	Female	4.87	1.36			
Task values	Male	5.10	1.31	1.579	0.12	0.003
	Female	4.94	1.37			
Sportspersonship attitude	Male	3.71	0.42	0.857	0.39	0.001
	Female	3.68	0.41			

**Table 5:** Expectancy beliefs, task values, and sportspersonship attitude according to age group.

Subscales		Mean	SD	*F*	*p*	Effect Size (*d*)
Expectancy beliefs	17–19 years	4.97	1.31	0.63	0.53	0.002
	15–16 years	4.91	1.31			
	12–14 years	4.83	1.42			
	Total	4.9	1.35			
Task values	17–19 years	5.07	1.33	4.19	0.01 *	0.011
	15–16 years	5.15	1.36			
	12–14 years	4.82	1.33			
	Total	5.02	1.35			
Sportspersonship attitude	17–19 years	3.71	0.4	0.94	0.94	0.003
	15–16 years	3.71	0.41			
	12–14 years	3.67	0.43			
	Total	3.7	0.42			

* *p* < 0.05.

**Table 6:** Post hoc Bonferroni test of task values between age groups.

Variables	µ Difference	*p*-Value	95% Confidence Interval for Difference	
			Lower Bound	Upper Bound
Task Values				
G1 versus G2	−0.08	1	−0.37	0.21
G1 versus G3	0.25	0.12	−0.04	0.55
G2 versus G3	0.33	0.02 *	0.05	0.62

Note. G1 = 17–19 years old; G2 = 15–16 years old; G3 = 12–14 years old, * *p* < 0.05.

**Table 7:** Pairwise comparison of male and female expectancy beliefs, task values, and sportspersonship attitude in each age group.

Age Group	Gender	µ Difference	*p*-Value	95% Confidence Interval for Difference	
				Lower Bound	Upper Bound
Expectancy Beliefs					
17–19 years	Male vs. Female	−0.26	0.14	−0.61	0.09
15–16 years	Male vs. Female	0.15	0.35	−0.17	0.48
12–14 years	Male vs. Female	0.34	0.05 *	−0.01	0.69
Task Values					
17–19 years	Male vs. Female	−0.07	0.69	−0.42	0.28
15–16 years	Male vs. Female	0.19	0.26	−0.14	0.51
12–14 years	Male vs. Female	0.42	0.02 *	0.07	0.77
Sportspersonship Attitude					
17–19 years	Male vs. Female	0.09	0.12	−0.02	0.19
15–16 years	Male vs. Female	0	0.99	−0.10	0.1
12–14 years	Male vs. Female	0.01	0.09	−0.10	0.12

* The mean difference is significant at the 0.05 level.

## Data Availability

The data is available upon request from the authors.

## References

[1] Vallerand R.J., Deshaies P., Cuerrier J.-P., Briere N.M., Pelletier L.G. (1996). Toward a multidimensional definition of sportsmanship. J. Appl. Sport Psychol..

[2] Kavussanu M., Boardley I.D. (2009). The prosocial and antisocial behavior in sport scale. J. Sport Exerc. Psychol..

[3] Vallerand R.J., Briere N.M., Blanchard C.M., Provencher P. (1997). Development and validation of the multidimensional sportspersonship orientations scale. J. Sport Exerc. Psychol..

[4] Kuan G., Abdullah N., Kueh Y.C., Ismail M., Shafei M.N., Morris T. (2019). Co-curricular activities and motives for participating in physical activity among health sciences students at Universiti Sains Malaysia, Malaysia. Malays. J. Med. Sci..

[5] Grasten A. (2015). Children’s expectancy beliefs and subjective task values through two years of school-based program and associated links to physical education enjoyment and physical activity. J. Sport Health Sci..

[6] Durik A.M., Shechter O.G., Noh M., Rozek C.S., Harackiewicz J.M. (2014). What if I can’t? Success expectancies moderate the effects of utility value information on situational interest and performance. Motiv. Emot..

[7] Wigfield A., Cambria J. (2010). Expectancy-value theory: Retrospective and prospective. The Decade Ahead: Theoretical Perspectives on Motivation and Achievement Advances in Motivation and Achievement. Adv. Motiv. Achiev..

[8] Gallardo L.O., Abarca-Sos A., Dona A.M. (2020). Expectancy-value model related to physical activity behaviors in Chilean and Spanish adolescents. Int. J. Environ. Res. Public Health.

[9] Kuan G., Roy J. (2007). Goal profiles, mental toughness and its influence on performance outcomes among Wushu athletes. J. Sports Sci. Med..

[10] Chriest A. (2017). Ice Hockey Coaches’ Beliefs and Perceptions of Coach Education. Ph.D. Thesis.

[11] Chan D.K.C., Hardcastle S., Dimmock J.A., Lentillon-Kaestner V., Donovan R.J., Burgin M., Hagger M.S. (2015). Modal salient belief and social cognitive variables of anti-doping behaviors in sport: Examining an extended model of the theory of planned behavior. Psychol. Sport Exerc..

[12] Adell F.L., Castillo I., Álvarez O. (2019). Personal and sport values, goal orientations, and moral attitudes in youth basketball. Rev. Psicol. Deporte.

[13] Fredericks J.A., Eccles J.S. (2002). Children’s competence and value beliefs childhood adolescence: Growth trajectories in two male-sex-typed domains. Dev. Psychol..

[14] Kren F., Kudlacek M., Wasowicz W., Groffik D., Fromel K. (2012). Gender differences in preferences of individual and team sports in Polish adolescents. Acta Gymnica.

[15] Hair J.F., Black W.C., Babin B.J., Anderson R.E. (2010). Multivariate Data Analysis: A Global Perspective.

[16] Chai S., Kueh Y.C., Yaacob N.M., Kuan G. (2019). Psychometric properties of the Malay version of the goal content for exercise questionnaire among undergraduate students at the Health Campus, Universiti Sains Malaysia. Malays. J. Med. Sci..

[17] Chin N.S., Teo E.W., Kuan G., Yi T. (2019). Adolescent athletes’ expectancy beliefs, task values and types of motivation in sports. Pertanika J. Soc. Sci. Humanit..

[18] Xiang P., McBride R., Guan J. (2004). Children’s motivation in elementary physical education: A longitudinal study. Res. Q. Exerc. Sport.

[19] Conley A.M. (2012). Patterns of motivation beliefs: Combining achievement goal and expectancy-value perspectives. J. Educ. Psychol..

[20] Pennington C.G. (2017). Moral development and sportsmanship in physical education and sport. J. Phys. Educ. Recreat. Danc..

[21] Rahimizadeh M., Arabnarmi B., Mizany M., Shahbazi M., Bidgoli Z.K. (2011). Determining the difference of aggression in male and female, athlete, and non-athlete students. Procedia–Soc. Behav. Sci..

[22] McPherson B.D., Curtis J.E., Loy J.W. (1989). The Social Significance of Sport–An Introduction to the Sociology of Sport.

[23] Lidor R., Ziv G. (2015). On-field performances of female and male field hockey players–A review. Int. J. Perform Anal Sport.

[24] Gurpinar B., Kursun S. (2013). Sportspersonship orientations of basketball and soccer players. Mediterr. J. Humanit..

[25] Mosquera R.P., Molinuevo J.S., Román I.R. (2007). Differences between international men’s and women’s teams in the strategic action of the penalty corner in field hockey. Int. J. Perform. Anal. Sport.

[26] Studer B., Knecht S. (2016). A benefit–cost framework of motivation for a specific activity. Prog. Brain Res..

[27] Altın M., Kıvrak A., Temür E., Tarman B., Serdar K., Denktaş M., Tüzer B., Kılıç M., Bozgüney R. (2019). Fair-play behavior of high school students playing futsal and football. J. Eurasia Sport Sci. Med..

[28] Goksel A.G., Zorba E. (2017). The examination of sportsmanship behaviors of beach handball players in turkey. Sport J..

[29] Karatas O., Savas B.G. (2019). Analysis on sportspersonship orientations of students studying in faculty of sports sciences/school of physical education and sports. Asian J. Educ. Train..

